# Tuning the fluorescence of Dy^3+^ via the structure of borophosphate glasses

**DOI:** 10.1038/s41598-023-28941-1

**Published:** 2023-02-02

**Authors:** Kristin Griebenow, Mai-Phuong Truong, Francisco Munoz, Robert Klement, Dusan Galusek

**Affiliations:** 1 Otto Schott Institute of Materials Research, Friedrich Schiller University Jena, Fraunhoferstrasse 6, 07743 Jena, Germany; 2 FunGlass, A. Dubček University of Trenčín, Študentská 2, 911 50 Trenčín, Slovakia; 3 Institute of Ceramics and Glass (CSIC), Kelsen 5, 28049 Madrid, Spain; 4 VILA-Joint Glass Centre of the IIC SAS, TnUAD, and FChPT STU, Študentská 2, 911 50 Trenčín, Slovakia

**Keywords:** Inorganic LEDs, Characterization and analytical techniques, Optical materials

## Abstract

The optical characteristics of Dy^3+^-doped phosphate and borophosphate glasses with different divalent network modifiers prepared by melt-quenching are studied. The glass sets (A) with a molar composition of 40MO–60P_2_O_5_ and (B) with a molar composition of 40MO–20B_2_O_3_–40 P_2_O_5_ are investigated, both with M = (Zn^2+^, Mg^2+^, Ca^2+^, Sr^2+^, or Ba^2+^) and all doped with 0.1 mol% Dy_2_O_3_. Raman and fluorescence spectroscopy are used to analyse the structure and optical characteristics of these glasses. Four typical Dy^3+^ emission bands in the yellow (572 nm), blue (483 nm) and red (633 and 752 nm) regions of the spectrum are observed in both sets. The fluorescence lifetimes in each glass set are correlated to the network modifier's ionic field strength. The Mg^2+^ and Zn^2+^ containing glasses have the longest fluorescence lifetimes. The yellow to blue emission intensity ratio of the respective bands can be used to indicate a symmetric environment around Dy^3+^ ions and varies with the ionic field strength of the modifier cations: a higher ionic field strength leads to a higher yellow to blue ratio, which in turn indicates a higher asymmetrical local coordination environment of Dy^3+^ ions in the glassy host network.

## Introduction

The three most often used types of interior lighting devices use incandescent lamps, fluorescent lamps, or light emitting diodes (LEDs). Particularly LEDs, which can be solid-state LEDs, organic LEDs, or polymer LEDs, have clear advantages over the other two such as a greater energy efficiency and a longer working life which result in a greater environmental friendliness. These advantages result from the spontaneous light emission in semiconductors caused by the radiative recombination of excess electrons and holes created in the presence of an electric current which resolves many constraining factors in other light sources. The nearly monochromatic emission of classic LEDs can be used to excite other phosphors which in return are able to emit white light. The combination of these two elements is the basis for "solid-state illumination" using white LEDs (WLEDs). The first commercial solid-state lighting WLED was produced in 1996 based on InGaN semiconductors and the inorganic phosphor (YAG:Ce). It opened the door for the further development of WLEDs^1^.

Rare earth (RE) ion-doped materials are widely used in photonic devices and other lighting applications because of their superior optical performance and a long and stable lifetime. WLEDs can be produced by using RE phosphors to generate blue or near-UV light and balancing it with a yellow emission or a complementary package of red, green, and blue emissions.

RE ion emission characteristics significantly depend on the electronic structure of the RE ion, the host matrix, the network modifiers and the doping concentration^2–4^. Among the RE ions in glass hosts, Dy^3+^ has a broad emission spectrum and a high luminescence efficiency. This is caused by two main emission bands in the blue and yellow regions of the visible spectrum, which correspond to the transitions ^4^F_9/2_ → ^6^H_15/2_ (magnetic dipole) and ^4^F_9/2_ → ^6^H_13/2_ (electric dipole). Due to a hypersensitivity of the yellow transition, its emission intensity is greatly impacted by the local field environment^5^, whereas the blue transition is less sensitive to the host. These emission bands enable to adjust the stimulation of white light by tuning the yellow to blue emission intensity ratio by changing the host composition. Furthermore, the ratio of the yellow to blue emission intensity is considered to be a measure for the Dy^3+^ environment distortion; a yellow to blue ratio ≥ 1 implies a high local symmetry around the Dy^3+^ ion^2,6^. It is possible to prolong the emission lifetime by increasing the yellow to blue ratio as well as the symmetric distortion of the host matrix^6^.

A phosphate glass network is composed of PO_4_ tetrahedral units which are connected by P-O covalent bonds. The degree of bonding varies with the composition of the glass^7^. The phosphate structures in the glass network are defined by the Q^*i*^ terminology, in which *i* represents the number of bridging oxygen atoms. Borate glasses, on the other hand, are composed of both trigonal BO_3_ and tetragonal BO_4_ units whose relative amounts strongly depend on the glass composition^8^. Adding B to the phosphate glass enables to obtain a variety of structural arrangements by linking [PO_4_] units with [BO_3_] and/or [BO_4_] units^7,9,10^. The potential of Dy^3+^ doping in different glass hosts has been demonstrated for borophosphate (BP), Li borate-, Sr-Li-Bi borate-, and fluorozirconate glasses^2,3,5,11–19^.

The aim of this study is to monitor the Dy^3+^ emission resulting from adding B_2_O_3_ to Dy^3+^-doped phosphate glasses and incorporate the divalent network modifiers Mg^2+^, Ca^2+^, Sr^2+^, Ba^2+^, or Zn^2+^.

## Experimental

### Glass preparation

Two glass sets were prepared by conventional melt-quenching using the chemicals H_3_BO_3_, NH_4_H_2_PO_4_ and ZnO (all from CentralChem, 99.5% purity), BaCO_3_ (AFT Bratislava, 98.5%), CaCO_3_ (CentralChem, 99.0%), MgCO_3_ (AFT Bratislava, 95.0%), Dy_2_O_3_ (Treibacher, 99.9%), and SrCO_3_ (Sigma-Aldrich, 99.9%). The molar composition of set (A) is 40 MO-60 P_2_O_5_ + 0.1 mol % Dy_2_O_3_ while the composition of set (B) is 40 MO-20 B_2_O_3_-40 P_2_O_5_ + 0.1 mol % Dy_2_O_3_, with M = Ba^2+^, Ca^2+^, Mg^2+^, Sr^2+^, or Zn^2+^. The nominal glass compositions are summarised in Table 1.

**Table 1:** Nominal glass compositions of the studied glasses. *Set (C) was studied earlier^2^ and the data are taken as comparison.

Set	MO (mol%) M = Mg^2+^, Ca^2+^, Sr^2+^, Ba^2+^ or Zn^2+^	B_2_O_3_ (mol%)	P_2_O_5_ (mol%)	Dy_2_O_3_ (mol%)
Set (A)	40	0	60	0.1
Set (B)	40	20	40	0.1
Set (C)*	40	10	50	0.1

Batches for 50 g of glass were homogenised for 1 h in a rotating homogenizer and melted in alumina crucibles using an electric furnace (Clare 4.0- Classic, Czech Republic). The batches were heated to 450 °C and kept there for 12 h. Subsequently the temperature was slowly increased to 1400 °C over the course of 7 h and held for 2 h to homogenize the melt. The melts were poured into graphite moulds and transferred to a furnace (LAC furnace, Ht60B controller, Czech Republic) preheated to a temperature of the corresponding glass transition temperature (T_g_) + 10 °C. The respective T_g_ were estimated based on previous research^2^. After 0.5 h, the furnace was switched off and the samples slowly cooled to room temperature (RT).

### Methods

Raman spectra were recorded from the bulk glasses with a Renishaw Raman Microscope inVia Reflex under 532 nm excitation using a Leica 50 × objective, an exposure time of 2 s and 10 accumulations.

The fluorescence spectra and luminescence decay curves were recorded at RT using a Fluorolog FL3-21 spectrometer (Horiba, France). Photoluminescence excitation (PLE) and emission spectra (PL) were measured in the front-face configuration at an excitation wavelength of 349 nm using a continuous Xenon lamp (450 W) as the excitation light source. Appropriate cut-off filters were used to eliminate the higher-order reflection artefacts in the photoluminescence spectra. The PL and the PLE spectra were measured under the same conditions, i.e. slit width, integration time and excitation/monitored wavelength. The PL spectra were corrected for the spectrometer optics and the excitation lamp response, whereas the PLE spectra were only corrected for the spectrometer optics.

Lifetime decay curves were obtained by exciting the samples using a Xenon flash lamp at 349 nm at RT and the blue (483 nm) and yellow (572 nm) emission wavelengths were monitored. The decay curves were normalized and fitted using a double-exponential function. All produced glasses contained a few bubbles and were colourless and transparent to the naked eye.

### Raman spectroscopy

**Figure 1:**
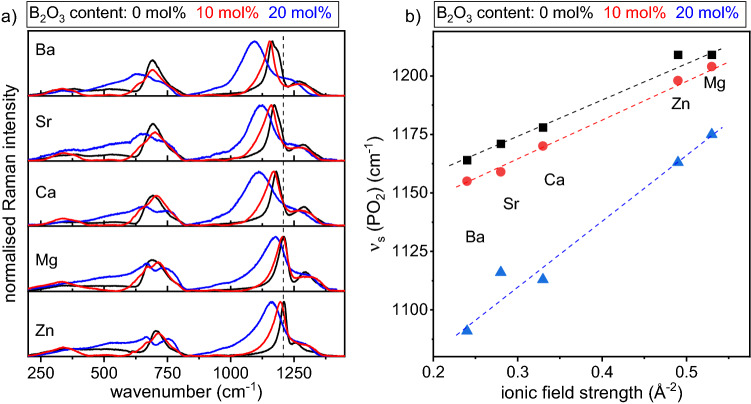
(**a**) Normalised Raman spectra of the glass set (A) in black and (B) in blue. The data for comparable spectra of the glass containing 10 mol% B_2_O_3_ are taken from Ref.^2^. (**b**) Raman shift of the ν_s_(PO_2_) as a function of the ionic field strength of the network modifier for the before mentioned three sets of glasses. All dashed lines are guides to the eye.

The normalised Raman spectra of the sets (A) and (B) with various network modifiers are presented in Fig. 1a alongside the comparably acquired spectra of a comparable glass containing 10 mol% B_2_O_3_^2^. The spectra of set (A) are dominated by two distinct bands at ~ 700 and 1200 cm^−1^ which can be assigned to the symmetric stretching vibrations of the bridging oxygen atoms connecting two PO_4_ tetrahedra (*ν*_s_(P-O-P)) and to the symmetric stretching vibrations of the phosphate Q^2^ groups (*ν*_s_(PO_2_))^7,20^. The minor feature at ~ 1270 cm^−1^ can be attributed to the asymmetric stretching vibrations of the non-bridging oxygen atoms of PO_2_ units^7^. The *ν*_s_(P-O-P) shifts to lower wavenumbers as the field strength of the network modifier decreases, i.e. from 705 cm^−1^ (Zn^2+^) to 690 cm^−1^ (Ba^2+^). The same trend is observed for the *ν*_s_(PO_2_), i.e. the vibration shifts from 1209 cm^−1^ (Zn^2+^) to 1163 cm^−1^ (Ba^2+^) accompanied by an increasing band width and a shoulder at ~ 1210 cm^−1^. These results are in good agreement with previous results on alkaline earth (AE) phosphate glasses^21–23^.

The Raman spectra become more complex when P_2_O_5_ is partially substituted by B_2_O_3_ as is discernible in Fig. 1a. The peak of the symmetric stretching vibrations of the phosphate Q^2^ groups (*ν*_s_(PO_2_)) shifts to lower wavenumbers when B_2_O_3_ is introduced, e.g. from 1209 cm^−1^ to 1161 cm^−1^ for Zn^2+^ containing glasses of set (A) and set (B), and increases its bandwidth, e.g. from 31 to 115 cm^−1^ (full width at half maximum) for the same glasses. This is due to a change in the next nearest neighbours of the Q^2^ groups and the formation of P^2^_1B_ and P^2^_2B_ structural units accompanied by an increasing variety of P-O bond angles and bond lengths^3,24–27^. The peak of the *ν*_s_(PO_2_) also shifts to lower wavenumbers as the ionic field strength of the network modifier decreases, i.e. 1161 (Zn^2+^), 1176 (Mg^2+^), 1108 (Ca^2+^), 1121 (Sr^2+^), and 1093 cm^−1^ (Ba^2+^) for glass set (B). The symmetric stretching vibration of P-O-P at around 700 cm^−1^ in the pure phosphate glasses is substituted by two overlapping bands at around 680 and 750 cm^−1^, which can be assigned to the P-O-B stretching vibrations^7^ and the breathing of borophosphate rings^28^. These bands are more distinct in the Mg^2+^ and Zn^2+^ containing glasses. The variation of the Raman shift within one kind of modifier is strongest for a B_2_O_3_ content of 20 mol% and the peak width also increases with a higher B_2_O_3_ content. The Raman shift of the maximum of the *ν*_s_(PO_2_) as a function of the ionic field strength of the network modifier is presented in Fig. 1b.

The ionic field strength of a cation is related to the ionicity of the cation-oxygen bond and can be calculated by Z/a^2^, where *Z* is the charge of the specific cation and *a* is the ionic distance for oxides^29^. The *ν*_s_(PO_2_) increases with increasing ionic field strengths and decreasing B_2_O_3_ contents. It is caused by the more covalent character of the P-O bonds, leading to an overall strengthened glass network.

### Fluorescence spectra and radiative properties

**Figure 2:**
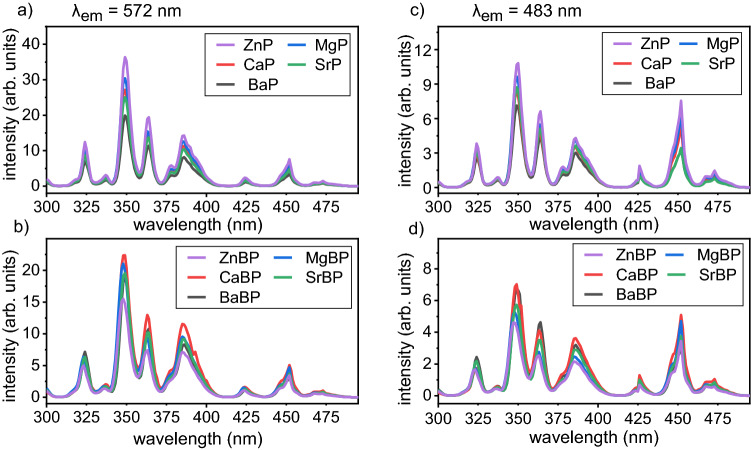
Excitation spectra monitored at 572 nm of (**a**) glass set (A) and (**b**) glass set (B) or monitored at 483 nm of (**c**) glass set (A) and (**d**) glass set (B).

The excitation spectra monitored at two prominent emission bands, 483 nm and 572 nm, are presented in the Fig. 2: the spectra of the pure phosphate glasses in Fig. 2a,c and the spectra of the BP glasses in Fig. 2b,d. They consist of eight electron transitions from the ground state ^6^H_15/2_ to the different excited states ^6^P_3/2_ (324 nm), ^4^I_9/2_ (337 nm), ^6^P_7/2_ (349 nm), ^6^P_5/2_ (364 nm), ^4^K_17/2_ (386 nm), ^4^G_11/2_ (425 nm), ^4^I_15/2_ (453 nm), and ^4^F_9/2_ (473 nm)^13,30^. The most intense band of all glasses at ≈349 nm is assigned to the ^6^H_15/2_ → ^6^P_7/2_ transition. Thus, this wavelength was used as an excitation wavelength for recording the emission spectra of both glass sets. The peak intensities vary if the network modifiers Mg^2+^, Ca^2+^, Sr^2+^, Ba^2+^, or Zn^2+^ are alternated while their positions (emission wavelengths) remain constant. The peak intensities also differ for the two host matrices: the peak intensity for glass set (A) decreases in the order Zn^2+^  > Mg^2+^  > Ca^2+^  > Sr^2+^  > Ba^2+^, while a different trend Ca^2+^  > Ba^2+^  > Sr^2+^  > Mg^2+^  > Zn^2+^ is observed for the glass set (B).

**Figure 3:**
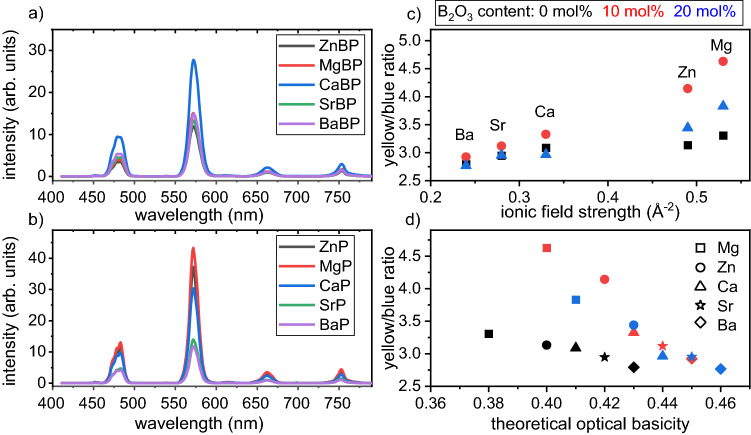
(**a**,**b**) Emission spectra of glass sets (A) and (B) excited at 349 nm. The luminescence intensity ratios of the investigated glass sets and a related glass set taken from Ref.^2^ are presented as a function of the ionic field strength of the network modifier (**c**) or as a function of the theoretical optical basicity of the glasses (**d**).

Figure 3a,b show the recorded emission spectra of Dy^3+^-doped glasses in the visible region of 400–800 nm. Four similarly shaped emission bands at ^4^F_9/2_ → ^6^H_15/2_ (483 nm), ^6^H_13/2_ (572 nm), ^6^H_11/2_ (663 nm) and ^6^H_9/2_ (752 nm) are observed for both glass sets. The ^4^F_9/2_ → ^6^H_13/2_ (yellow) and ^4^F_9/2_ → H_15/2_ (blue) transitions are the two most intense and respectively correspond to the electric or magnetic dipole transitions. In general, the yellow emission at 572 nm is roughly four times as intense as the blue emission at 483 nm, particularly when excited with a wavelength of 349 nm.

The blue transition with its magnetic dipole character is not significantly affected by the local environment around the Dy^3+^ ion, while the yellow emission is hypersensitive and strongly influenced by the local environment of Dy^3+^. The yellow to blue intensity ratio can be used as a spectroscopic reference to probe the local symmetry around Dy^3+^ and the covalency of the Dy-O bonds^31,32^, as a higher asymmetry should increase the transition probabilities of the hypersensitive bands. A high yellow to blue ratio results from an increased local asymmetry and a high covalency of the Dy-O bond. Figure 3c,d present the yellow to blue ratios of the investigated glasses including results from Ref.^2^.

These results show that the yellow to blue ratio increases with the increasing ionic field strength of the network modifier and has its maximum in the Mg^2+^ containing glasses. It increases slightly when modifiers with a low ionic field strength occur in the matrix, ranging from ~ 2.75 to ~ 3.25 between Ba and Ca, respectively. Larger changes in the yellow to blue ratio are observed for the high field strength ions Mg^2+^ and Zn^2+^. The yellow to blue ratio initially increases with the substitution of P_2_O_5_ by B_2_O_3_ and shows a maximum at 10 mol % B_2_O_3_. This is the opposite yellow to blue ratio trend of that reported for the effect of different AE ions on the luminescence properties of Dy^3+^ in glasses with the general molar composition 50Li_2_O-20MO-29.5B_2_O_3_-0.5Dy_2_O_3_ and M = Mg^2+^, Ca^2+^, Sr^2+^ or Ba^2+^ where Ba^2+^ showed the highest and Mg^2+^ the lowest yellow to blue ratio^3^. It was concluded that the covalency degree of the Dy-O bond increases with the atomic weight of the AE ion based on a Judd–Ofelt analysis^3^. This disagreement to the results presented here could be caused by the differing glass-forming system as well as the modifier types and contents. Any change in the composition of the glass system and the structure of the network would thus have a direct effect on the Dy^3+^ ion surroundings.

In order to reach a common basis for the elucidation of the yellow to blue ratio behaviour, the dependency of the yellow to blue ratio to the calculated optical basicity of the glasses will now be explored. The evolution of the yellow to blue ratio over the theoretical optical basicity Λ_th_ of the host matrix is presented in Fig. 3d, where the Λ_th_ values were calculated according to Eq. (1):

1 $${\Lambda }_{th}={\mathrm{\rm X}}_{A{O}_{a/2}}\bullet\Lambda \left(A{O}_{a/2)}\right)+ {\mathrm{\rm X}}_{B{O}_\frac{b}{2}}\bullet\Lambda \left(B{O}_{b/2)}\right) +\dots$$

with Λ(AO_a/2_), Λ(BO_b/2_), … representing the optical basicity of the oxides AO_a/2_, BO_b/2_, … and Χ_AOa/2_, Χ_BOb/2_, … the equivalent fractions, i.e. the proportion of oxygen atoms they contribute to the network^33–35^. Λ_th_ increases when P_2_O_5_ is substituted by B_2_O_3_, e.g. in Mg^2+^ containing glasses from 0.38 (pure phosphate glass) to 0.41 (20 mol% B_2_O_3_), and increases within one set of glasses when the ionic field strength of the network modifier decreases, e.g. in set (A) from 0.38 (Mg^2+^) to 0.43 (Ba^2+^). The trend of the yellow to blue ratio over Λ_th_ is almost linear within each glass set for Ba^2+^, Sr^2+^ and Ca^2+^ but changes significantly when Mg^2+^ or Zn^2+^ are present, indicating a decreasing electron donor power of the host matrix with a high field strength of the network modifiers.

**Figure 4:**
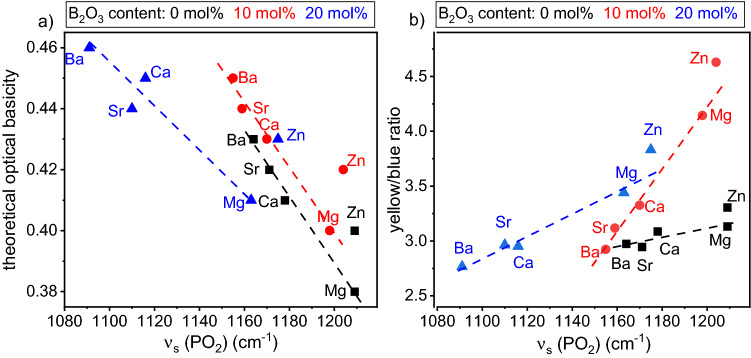
Theoretical optical basicity (**a**) and the yellow to blue ratio (**b**) of the glass sets (A) and (B) as well as the data of Ref.^2^ as a function of the ν_s_(PO_2_) extracted from the respective Raman spectra.

The correlation of the Λ_th_ and the yellow to blue ratios with the information obtained from the Raman spectra are presented in Fig. 4. Figure 4a shows the Λ_th_ over the Raman shift of all glass sets and the yellow to blue ratio obtained from the emission spectra of the glasses are presented in Fig. 4b. Λ_th_ decreases linearly with an increasing Raman shift of the *ν*_s_(PO_2_) (and increasing ionic field strength of the network modifier) within the AE ions, but the Zn^2+^ containing glasses do not follow this trend. The yellow to blue ratio increases linearly with an increasing Raman shift of the *ν*_s_(PO_2_). The Zn^2+^ containing glasses again do not follow the described trend.

### Luminescence lifetime

**Figure 5:**
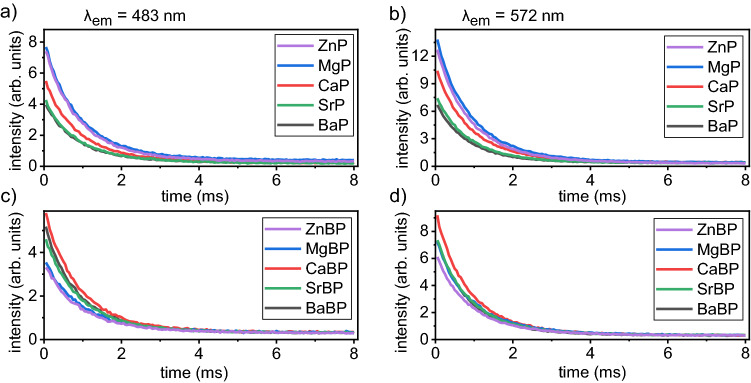
Luminescence decay curves for λ_emission_ = 483 nm of (**a**) set (A) and (**c**) set (B) as well as λ_emission_ = 572 nm of (**b**) set (A) and (**d**) set (B).

The luminescence decay curves of set (A) are presented in Fig. 5a,b, whereas the decay curves of set (B) are presented in Fig. 5c,d. They correspond to the ^4^F_9/2_ → ^6^H_13/2_ (572 nm) and ^4^F_9/2_ → ^6^H_15/2_ (483 nm) transitions and were fitted using the bi-exponential function Eq. (2)^36–39^:

2 $$I(t)={I}_{0}+{A}_{1}\mathit{exp}\left(\frac{t}{{\tau }_{1}}\right)+{A}_{2}\mathit{exp}\left(\frac{t}{{\tau }_{2}}\right)$$

where I_0_ is the fluorescence intensity at t = 0, τ_1_ and τ_2_ represent the luminescence lifetimes and A_1_ and A_2_ are the respective weighing parameters. The average experimental lifetime (τ_exp_) is calculated according to Eq. (3)^36–39^:

3 $${\tau }_{exp}=\frac{{A}_{1}{\tau }_{1}^{2}{+A}_{2}{\tau }_{2}^{2}}{{A}_{1}{\tau }_{1}{+A}_{2}{\tau }_{2}}$$

All lifetime values and weighting factors of the analysed glasses are listed in Table 2.

**Table 2:** Measured (τ_1, 2_) and calculated (τ_exp_) lifetime values of the emission λ_emission_ = 483 nm and λ_emission_ = 572 nm of the Dy^3+^ ions in the investigated glass sets measured under λ_excitation_ = 349 nm.

Glass	Em 483 nm					Em 572 nm				
	τ_1_	τ_2_	τ_exp_	A_1_	A_2_	τ_1_	τ_2_	τ_exp_	A_1_	A_2_
	(ms)	(ms)	(ms)			(ms)	(ms)	(ms)		
ZnP01Dy	0.63	0.63	0.63	364.030	363.869	0.35	0.78	0.7	431.606	878.946
MgP01Dy	0.24	0.72	0.69	147.583	628.184	0.23	0.73	0.69	258.530	1159.538
CaP01Dy	0.36	0.83	0.72	218.739	334.769	0.31	0.73	0.67	273.562	779.380
SrP01Dy	0.32	0.85	0.74	173.778	252.218	0.38	0.77	0.67	308.936	439.708
BaP01Dy	0.39	0.79	0.68	169.483	225.743	0.3	0.65	0.62	138.442	530.522
ZnBP01Dy	0.43	0.96	0.78	172.329	151.826	0.41	0.88	0.73	302.920	311.965
MgBP01Dy	0.45	0.89	0.76	151.255	186.924	0.25	0.76	0.71	188.807	550.838
CaBP01Dy	0.41	0.79	0.69	231.485	340.924	0.35	0.76	0.67	347.166	579.887
SrBP01Dy	0.44	0.82	0.70	201.602	249.799	0.35	0.73	0.67	228.186	519.752
BaBP01Dy	0.31	0.73	0.67	149.786	364.230	0.37	0.74	0.65	291.545	457.924

A_1,2_ are the respective weighting factors obtained from the Eqs. (1) and (2).

The decay curves of the two highest emission intensities at 483 and 572 nm show that the ionic field strength impacts the emission lifetime. The Mg^2+^ or Zn^2+^ containing pure phosphate glasses show the highest emission intensity, followed by the Ca^2+^, Sr^2+^ and Ba^2+^ containing glasses. The intensities are highest for Ca^2+^ and lowest for Zn^2+^ or Mg^2+^ in the case of glass set (B). The overall trends are the same for both emission intensities.

**Figure 6:**
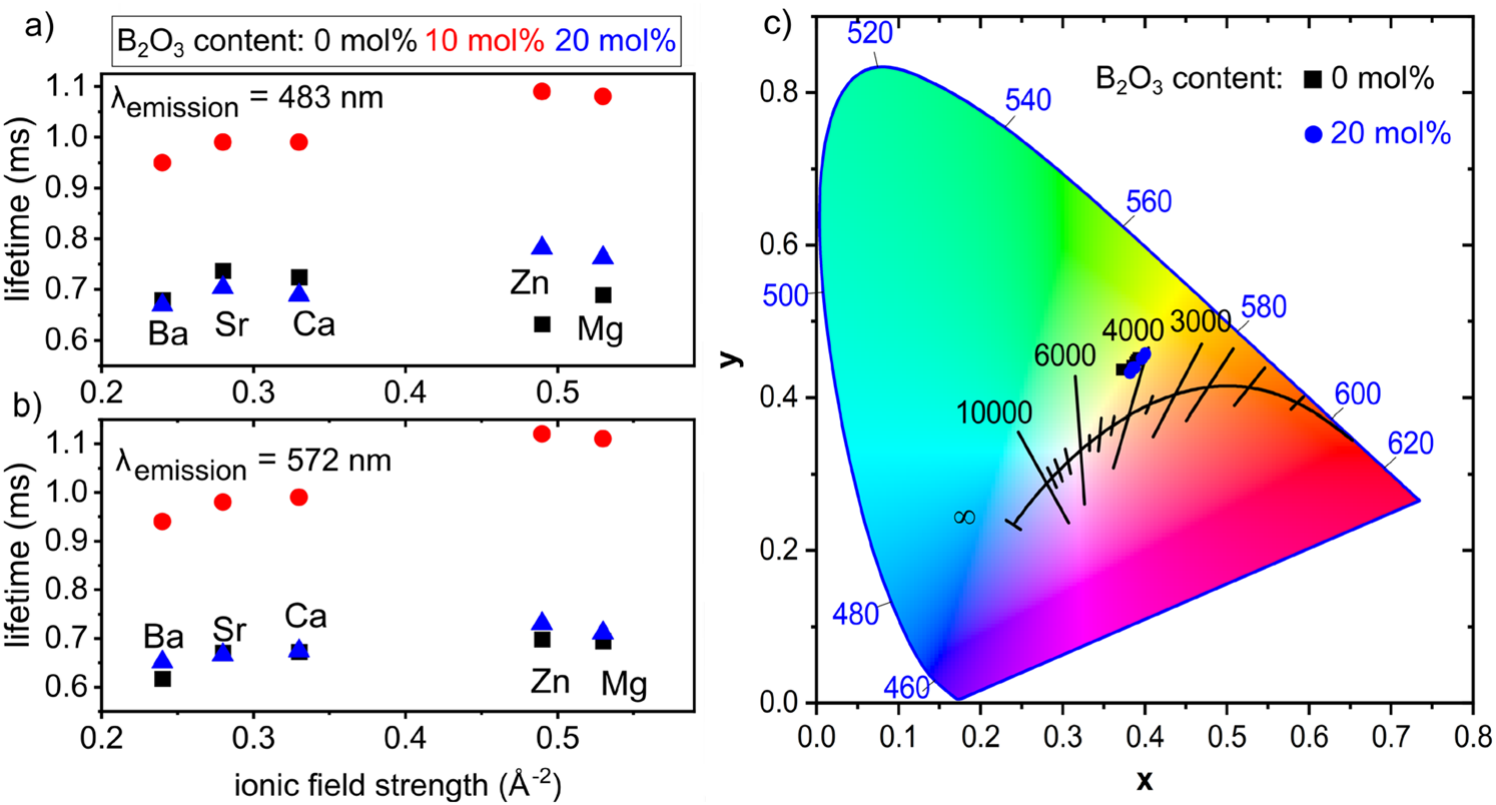
Experimental lifetime values of the (**a**) 483 nm and (**b**) 572 nm emission versus the ionic field strength of the respective network modifier. (**c**) CIE 1931 chromaticity coordinates of the emission of the Dy^3+^ doped glasses of set (A) and (B) under 349 nm excitation.

Figure 6a,b presents the calculated lifetimes over the respective ionic field strength of the network modifier for the 483 or 572 nm emission. The emissions of Dy^3+^ ions in the Mg^2+^ or Zn^2+^ containing BP glasses of set (B) have a longer lifetime than those in the pure phosphate glasses of set (A). However, low ionic field strength glasses containing the modifiers Ba^2+^, Sr^2+^ or Ca^2+^ show longer light emissions in set (A) than in set (B). Divalent metal ions have a significant effect on the luminescence lifetimes of glass products because of their different ionic field strengths. Higher cationic field strengths lead to more stable M–O bonding and hence a longer emission lifetime of the active ion (Dy^3+^). This trend is better observed for high ionic field strength modifiers (such as Mg^2+^ and Zn^2+^) than for the lower ionic field strength modifiers Ca^2+^, Sr^2+^ or Ba^2+^. However, the results presented above do not show a clear trend in the emission profiles when changing M^2+^ ions within a glass set.

The obtained lifetime values of this study are in good agreement with literature data summarised in Table 3. Fluoride containing glasses generally have longer lifetimes because of their lower phonon lattice energy. This is reflected in the comparably long emission lifetime in Ref.^11^. Shamshad et al. investigated the influence of different AE ions on the Dy^3+^ emission properties in Li borate glasses^3^ and found an opposite correlation of ionic field strength and emission lifetime: to the best of our knowledge, they measured the longest lifetime in the Ba^2+^ containing glass. Their comparably short lifetimes might be caused by concentration quenching, as they used a doping concentration of 0.5 mol% Dy_2_O_3_. A previous study on BP glasses with the general composition 40MO-10B_2_O_3_-50P_2_O_5_ found that a similar trend exists for the correlation of the Dy^3+^ emission lifetime with the ionic field strength of the network modifier^2^. These values are included in Fig. 6a,b for comparison. Lifetimes of up to 1.1 ms were measured for the Zn^2+^ containing glass^2^. Considering these previous results enables to conclude that the emission lifetime increases when P_2_O_5_ is partially substituted by B_2_O_3_ and then decreases again, when the boron content exceeds a certain threshold.

**Table 3:** Experimental lifetimes of the 572 nm emission of Dy^3+^-doped glasses taken from the literature.

Glass composition (mol %)	Temperature	τ_exp_ (ms)	References
57ZrF_4_-34BaF_2_-4.9LaF_3_-4AlF_3_-0.1DyF_3_	294 K	1.033	^11^
25BaO-5B_2_O_3_-69.8P_2_O_5_-0.2Dy_2_O_3_	RT	0.73	^13^
50Li_2_O-20MgO-29.5B_2_O_3_-0.5Dy_2_O_3_	RT	0.523	^3^
50Li_2_O-20CaO-29.5B_2_O_3_-0.5Dy_2_O_3_	RT	0.551	^3^
50Li_2_O-20SrO-29.5B_2_O_3_-0.5Dy_2_O_3_	RT	0.563	^3^
50Li_2_O-20BaO-29.5B_2_O_3_-0.5Dy_2_O_3_	RT	0.6	^3^

As discussed above, the hypersensitive transition in the host matrix is considered to be an indicator of the environment around the Dy^3+^ ion. It is well described by the yellow to blue ratio, where the yellow intensity varies with the electronic field strength but the blue intensity, affected by the magnetic field strength, remains nearly constant. Furthermore, the yellow to blue ratio can be a measure of the strength of the covalent or ionic bonding between the Dy^3+^ ions and the surrounding ligands^3,31^. As the yellow to blue ratio increases with increasing ionic field strength, the covalent character of the Dy-O bond also increases. This increase is present in all glass sets of this study and is independent of the B_2_O_3_ content. Figure 1b shows that the Raman shift of [PO_2_] groups increases when network modifiers with a high ionic field strength are present, indicating that the glass network becomes strengthened. The highest shift is observed for Mg^2+^ containing glasses which also show the highest values for the yellow to blue ratio, proving that the P-O bond strength directly influences the Dy-O bonds and thus the emission properties of the Dy^3+^ ions.

### Color coordinates

The emission color coordinates of the Dy^3+^ doped P and BP glasses under 349 nm excitation were calculated from the emission spectra and are summarised in Table 4. The obtained values are plotted onto the framework of the CIE 1931 chromaticity diagram in Fig. 6c, whose standard equal energy point (x = 0.33, y = 0.33) corresponds to the white light emission. The emission colour coordinates of the studied glasses are near the centre and vary from 4020 to 4523 K, i.e. they are in the “cool” white light emission range between 4000 and 6500 K. Despite their different glass compositions, the investigated glasses exhibit relatively small variations in their colour coordinates which are in between the colour coordinates of fluorescence tubes (~ 3935 K) and day light (~ 5500 K): they show slightly higher values than warm white light (< 4000 K). Hence these glasses are promising materials for white light applications and their emission colour can be controlled by choosing a suitable network modifier for tuning the yellow to blue ratio.

**Table 4:** Calculated colour coordinates and their temperatures (CCT) of the Dy^3+^ emission under 349 nm excitation.

Glass	x	y	CCT (K)
MgP01Dy	0.39	0.45	4149
CaP01Dy	0.39	0.45	4207
SrP01Dy	0.38	0.44	4269
BaP01Dy	0.37	0.44	4523
ZnP01Dy	0.39	0.45	4181
MgBP01Dy	0.40	0.46	4020
CaBP01Dy	0.39	0.44	4198
SrBP01Dy	0.39	0.44	4248
BaBP01Dy	0.38	0.43	4289
ZnBP01Dy	0.40	0.45	4075

## Conclusions

The structure of Dy^3+^ containing phosphate and borophosphate glasses was investigated and the emission characteristics of the Dy^3+^ ion were analysed. The Raman spectra reveal a progressive structural modification when B is introduced into the phosphate glass network. Under the 349 nm excitation, Dy^3+^ emits four typical bands in both glass environments. With increasing ionic field strength of the network modifier, the yellow to blue emission intensity ratio also increases. Substituting P_2_O_5_ by B_2_O_3_ leads to a progressive distortion of the local symmetry around the Dy^3+^ ions and causes not only increasing yellow to blue ratios but also longer emission lifetimes. Beyond a certain content, B_2_O_3_ in the glasses enables formation of P-O-B bonds in the network, which create a more symmetric coordination environment around the Dy^3+^ ions.

## Data Availability

The datasets used and/or analysed during the current study are available from the corresponding author on reasonable request.
